# Selective monitoring of the protein-free ADP-ribose released by ADP-ribosylation reversal enzymes

**DOI:** 10.1371/journal.pone.0254022

**Published:** 2021-06-30

**Authors:** Samuel Kasson, Nuwani Dharmapriya, In-Kwon Kim

**Affiliations:** Department of Chemistry, University of Cincinnati, Cincinnati, OH, United States of America; University of South Alabama Mitchell Cancer Institute, UNITED STATES

## Abstract

ADP-ribosylation is a key post-translational modification that regulates a wide variety of cellular stress responses. The ADP-ribosylation cycle is maintained by writers and erasers. For example, poly(ADP-ribosyl)ation cycles consist of two predominant enzymes, poly(ADP-ribose) polymerases (PARPs) and poly(ADP-ribose) glycohydrolase (PARG). However, historically, mechanisms of erasers of ADP-ribosylations have been understudied, primarily due to the lack of quantitative tools to selectively monitor specific activities of different ADP-ribosylation reversal enzymes. Here, we developed a new NUDT5-coupled AMP-Glo (NCAG) assay to specifically monitor the protein-free ADP-ribose released by ADP-ribosylation reversal enzymes. We found that NUDT5 selectively cleaves protein-free ADP-ribose, but not protein-bound poly- and mono-ADP-ribosylations, protein-free poly(ADP-ribose) chains, or NAD^+^. As a *proof-of-concept*, we successfully measured the kinetic parameters for the exo-glycohydrolase activity of PARG, which releases monomeric ADP-ribose, and monitored activities of site-specific mono-ADP-ribosyl-acceptor hydrolases, such as ARH3 and TARG1. This NCAG assay can be used as a general platform to study the mechanisms of diverse ADP-ribosylation reversal enzymes that release protein-free ADP-ribose as a product. Furthermore, this assay provides a useful tool to identify small-molecule probes targeting ADP-ribosylation metabolism and to quantify ADP-ribose concentrations in cells.

## Introduction

ADP-ribosylation is a reversible post translational modification (PTM) that regulates many cellular signaling pathways, including DNA repair, DNA replication, gene expression, and cell death [1–4]. The ADP-ribosylation cycle is a tightly controlled process involving a variety of different enzymes classified as ADP-ribose writers that add monomeric or polymeric ADP-ribose units, readers that specifically interact with ADP-ribose units to mediate cellular signaling, and erasers that cleave poly- or site-specific mono-ADP-ribosylations [5–10].

The homeostasis of poly(ADP-ribosyl)ation (PARylation), the most widely studied form of ADP-ribosylation, is maintained through the interplay between two predominant enzymes, poly(ADP-ribose) polymerase 1 (PARP1) and poly(ADP-ribose) glycohydrolase (PARG) [11–13]. Upon DNA damage, PARP1 becomes enzymatically activated and incorporates polymeric ADP-ribose chains onto itself and target proteins, using NAD^+^ as a substrate, to signal DNA damage. However, uncontrolled accumulation of PAR is cytotoxic, leading to the PAR-dependent cell death (called parthanatos) by triggering the mitochondrial release of apoptosis-inducing factor (AIF) [14–16]. Therefore, cellular level of PARylation is tightly controlled by PARG. The importance of PAR turnover is further highlighted by the embryonic lethal phenotype found in *PARG*^–/–^mice [17].

PARG has both endo- and exo-glycohydrolase activity that specifically cleave the internal and terminal sites of PAR, respectively [18–20]. Although PARG releases oligo(ADP-ribose) chains in early stage of reactions [18–20], it is believed that the primary enzymatic products of PARG is monomeric ADP-ribose [21,22]. Furthermore, PARG is unable to digest the last ADP-ribose unit attached to proteins, which is the substrate for site-specific mono-ADP-ribosyl (MAR) hydrolases, including MacroD1/D2 (Asp/Glu-MAR) [23,24], ARH3 (Ser-MAR) [25], and ARH1 (Arg-MAR) [26,27]. In all these cases, including the exo-glycohydrolase activity of PARG, protein-free mono-ADP-ribose is the final reaction product released by ADP-ribosyl-acceptor hydrolase activities.

Historically, the mechanisms of erasers of site-specific ADP-ribosylations have been underexplored, compared to writers of ADP-ribosylations. This could in part be attributed to the lack of quantitative and convenient tools that measure the reversal of site-specific mono-ADP-ribosylations. The most widely used method, an enzymatic ADP-ribosylation using ^32^P-NAD^+^ as a substrate for ADP-ribosyl transferases, is highly cumbersome [28,29]. Efforts have also been made in the development of a fluorescence-based assay [30,31], but this also has limitations in that it is unable to measure the release of ADP-ribose from ADP-ribosylated proteins. Very recently, a chemical conversion of α-NAD^+^ into fluorescent compound was adopted to monitor the activity of mono-ADP-ribosyl hydrolases [32], but this assay format can only be applied to enzymes that can hydrolyze α-NAD^+^. Although we previously developed a HPLC-coupled biochemical system that can monitor the release of oligo(ADP-ribose) [33], the ADP-ribosylation field still lacks tools that conveniently and selectively measure the release of protein-free ADP-ribose that is a product of functionally diverse PAR- and MAR hydrolases in nature.

**Fig 1:**
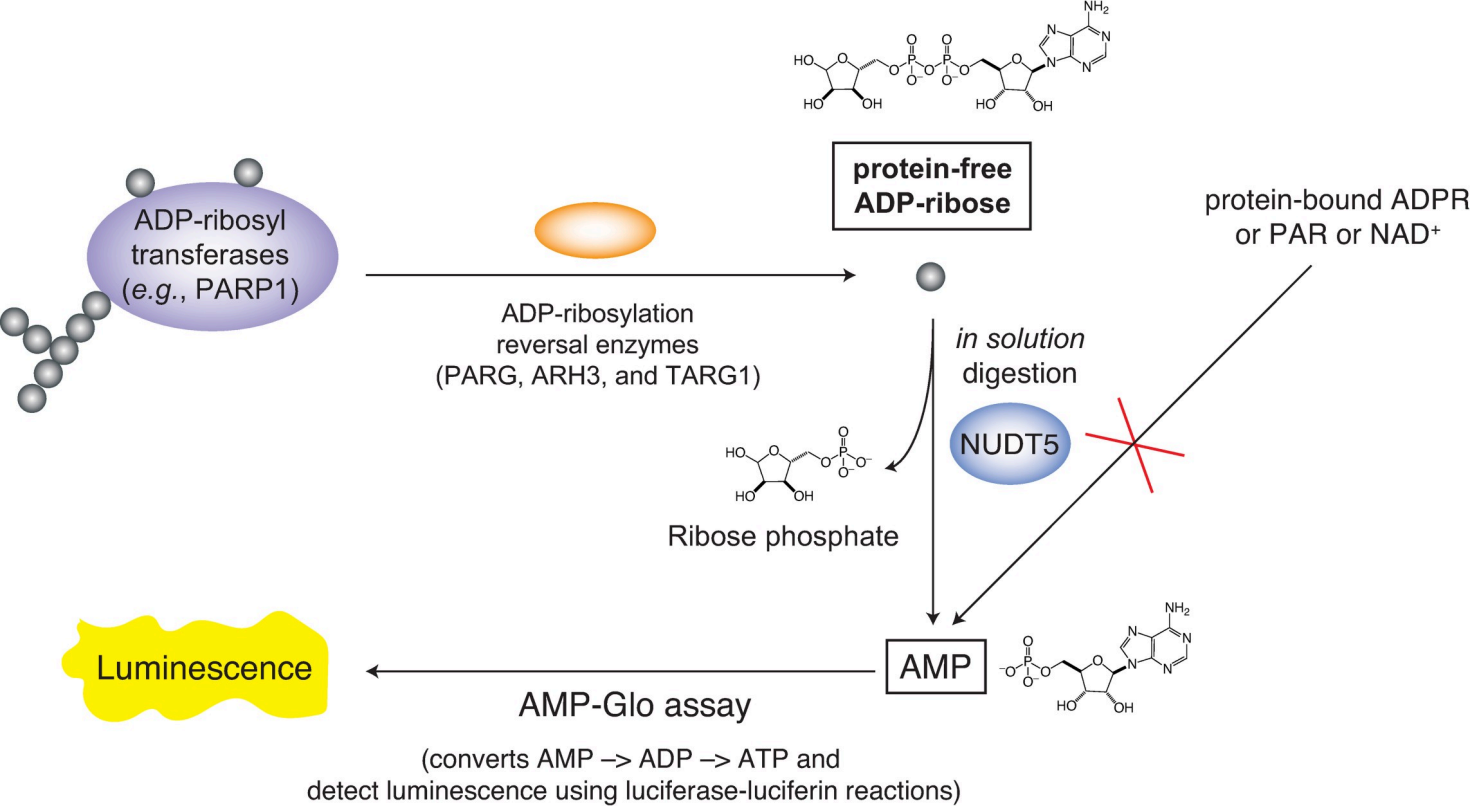
A scheme for the NUDT5-coupled AMP-Glo (NCAG) assay. NUDT5 has a unique substrate selectivity and can selectively cleave the protein-free mono-ADP-ribose into AMP and ribose phosphate, but not protein-bound ADP-ribosylations or protein-free poly(ADP-ribose) or NAD^+^. The cleaved AMP is subsequently detected by the AMP-Glo assay.

Here, using the unique substrate specificity of NUDT5, we have developed a novel quantitative assay platform that can selectively monitor the protein-free mono-ADP-ribose released by ADP-ribosylation reversal enzymes (Fig 1). We have shown that NUDT5 has strict substrate selectivity to the protein-free monomeric ADP-ribose. By coupling NUDT5 and the AMP-Glo assay, which quantitatively measures the AMP concentrations [34], we calculated the concentrations of ADP-ribose in the substrates that are cleavable by PARG. Furthermore, we successfully measured the kinetic parameters of the exo-glycohydrolase activity of PARG, which releases protein-free ADP-ribose. Collectively, this novel NUDT5-coupled AMP-Glo (NCAG) assay format will provide a new toolkit to selectively monitor the protein-free ADP-ribose either released by diverse ADP-ribosyl-acceptor hydrolases or as cellular responses to genotoxic stresses in cells.

## Materials and methods

### Plasmids and protein purification

The human NUDT5 gene was synthesized (Geneuniversal. Inc) and then cloned into a modified pET21b vector with an N-terminal His6 tag and a following cleavage site for PreScission protease (pET21b-His6-pps). The pET21b-His6-pps-NUDT5 was introduced into *E*. *coli* BL21(DE3) cells harboring a pKJE7 plasmid expressing chaperones (Takara) and cells were grown on LB agar plates containing 100 μg/ml Ampicillin and 34 μg/ml Chloramphenicol. After overnight incubation at 37°C, a single colony was selected from the plate and added to a 100 mL seed culture of LB media with 100 μg/ml Ampicillin and 34 μg/ml Chloramphenicol which was grown overnight at 37°C. Then, 20 mL of seed culture was added to 1 L of LB media with 100 μg/ml Ampicillin and 34 μg/ml Chloramphenicol and cells were grown at 37°C until OD_600_ reaches ~ 0.7. At this point, cells were induced by adding 1 mM isopropyl-beta-D-thiogalactoside (IPTG) and 1.6 mg/ml L-arabinose (for overexpression of chaperones) overnight at 16°C. NUDT5 was purified by affinity capture on a Ni-NTA (GE Healthcare) column in 50 mM Tris-HCl, 0.1 M NaCl pH 7.5 (Buffer A). After washing with 5 CV (column volumes) of Buffer A containing 10 mM imidazole and 5 CV of Buffer A containing 20 mM imidazole, the protein was eluted in 5 CV of Buffer A containing 250 mM imidazole. The protein was then loaded onto a QFF anion exchange column (GE Healthcare) and eluted with a NaCl gradient (0.1–1 M) in 50 mM Tris-HCl pH 7.5. Fractions with NUDT5 were pooled and PreScission protease was added to cleave the N-terminal histidine tag overnight at 4°C. Finally, NUDT5 was loaded to a Superdex 200 (GE Healthcare) size-exclusion column using a buffer containing 25 mM Tris-HCl pH 7.5, 150 mM NaCl, 1 mM dithiothreitol (DTT, Goldbio), and 5% glycerol. Human NUDT16 gene was synthesized (Geneuniversal. Inc) and then cloned into pET21b-His6-pps. NUDT16 was purified using the same protocol as NUDT5 as described above.

For PARylation of PARP1, the DNA-binding domain (DBD; residues 1–374) domain and the PARP1C catalytic domain (PARP1C; residues 375–1014) of human PARP1 were purified as described previously [35]. The catalytic domain of human PARG (hPARG^389^, residues 389–976), was purified as described previously [33]. Proteins samples were then aliquoted out, flash frozen with liquid nitrogen, and finally stored at– 80°C. A gene for human HPF1 was synthesized and cloned into the pET21b-His6-pps by Geneuniversal. Inc. HPF1 was purified as described previously [36].

For western blotting, to monitor the protein ADP-ribosylations, AF1521-Fc (anti-pan-ADPR antibody) was purified as described previously [37] (Dr. W. Lee Kraus kindly provided a pET19b-Af1521-Fc plasmid).

### Preparation of PARylated, Ser-MARylated, and Asp/Glu-MARylated PARP1

PARylated PARP1 was prepared as described previously [35,38]. Briefly, PARP1C (2 μM), DBD (2 μM), nicked double stranded DNA (2 μM), and NAD^+^ (2 mM) (Sigma) were mixed and incubated for 1 hr at 37°C in 50 mM HEPES pH 8.0 and 10 mM MgCl_2_. Then, PARylated PARP1 was desalted using a PD-10 (GE healthcare) column in a buffer containing 25 mM Tris-HCl (pH 7.5), 50 mM NaCl, and 0.01% NP-40.

To prepare Asp/Glu-MARylated PARP1, PARylated PARP1 was incubated with 10 μM Olaparib (Seleckchem) and 0.62 μM PARG for 1 hour at 37°C. This reaction was then desalted as above with using a PD-10 (GE Healthcare) column in a buffer containing 25 mM Tris-HCl (pH 7.5), 50 mM NaCl, and 0.01% NP-40.

To prepare Ser-MARylated PARP1, PARP1 was PARylated in the presence of 2 μM HPF1. Then Ser-MARylated PARP1 was prepared in the same way as Asp/Glu-MARylated PARP1. All protein aliquots were stored at– 80°C.

### Preparation of protein-free PAR chains

Poly(ADP-ribose) chains were prepared using the protocol designed by Tan, *et*. *al*. [39]. Briefly, PAR chains were synthesized on a 20 mL reaction scale using 50 mM Tris-HCl (pH 7.5), 10 mM MgCl_2_, 1 mM DTT, 12.5 μg/mL calf thymus DNA, 2 μM full-length PARP1, 4 μM PARP1-C, and 2 mM NAD^+^. PARylated PARP1 was then precipitated using 10% ice-cold TCA and centrifuged at 14,000 rpm for 10 minutes. The PARylated PARP1 pellet was then resuspended in 1 M KOH and 50 mM EDTA and incubated in this solution for 1 hour at 60°C. PAR chains were then purified with a dihydroxyboryl Bio-Rex 70 column [38]. Briefly, 250 mM ammonium acetate pH 9.0 with 6 M guanidine hydrochloride and 20 mM EDTA was added until the pH reached 9.0. The sample was then added to a dihydroxyboryl Bio-Rex 70 column (PAR-affinity column) which had been equilibrated in the same buffer. The column was then washed with 1 M ammonium acetate pH 9.0 before elution with water. Next, the sample was size-fractionated on a DNA-Pac PA100 (Dionex) ion-exchange column. Size-fractionated pools, determined from elution peaks, were desalted with water using a PD-10 desalting column before being vacuum dried and redissolved into water at a concentration of ~ 1 mM determined from the extinction coefficient at 260 nm (13500 cm^-1^ M^-1^) [39].

### Western blotting

Digestions of PARylated, Serine MARylated, and Aspartate/Glutamate MARylated PARP1 were performed using 5 μM NUDT16, ARH3, and TARG1 respectively as well as with NUDT5. Reactions were done for 1 hour at 37°C in a NCAG buffer (100 mM Tris-HCl pH 7.0 with 10 mM MgCl_2_ and 2 mM DTT). Reactions were stopped with the addition of 4X SDS-loading dye (Bio-Rad) and boiled for 30 seconds. Samples were loaded onto a Mini-PROTEAN TGX Gel and run until completion. Samples were then transferred to polyvinylidene fluoride (PVDF) membrane and membranes were immediately incubated in a blocking buffer (5% dry milk, 137 mM NaCl, 2.7 mM KCl, 10 mM Na_2_HPO_4_, 1.8 mM KH_2_PO_4,_ and 0.1% Tween-20 pH 7.4) for one hour at room temperature. Membranes were then incubated with anti-pan-ADPR antibody (AF1521-Fc, 3 μg/ml final concentration) for 1 hour at room temperature in a blocking buffer. Membranes were then rinsed once with PBST before doing three washes with PBST for 10 minutes each at room temperature. After washing, membranes were incubated at room temperature for 30 minutes with 0.267 mg/mL Alexa Fluor488 goat anti-rabbit IgG secondary antibody (Invitrogen) in a blocking buffer. Afterward, membranes were rinsed quickly with PBST before being washed three times with PBST as before. The blot was then visualized the iBRIGHT-FL1000 imager (ThermoFisher). After antibody imaging, membranes were incubated in Ponceau S staining solution (0.1% Ponceau S in 5% acetic acid) for 30 seconds at room temperature before several rinses with DI water and being left to air dry.

### NUDT5-coupled AMP-Glo (NCAG) assay

All AMP-Glo assays were done in a 384 well microplate (Corning, Inc) in triplicate using an AMP-Glo Assay kit (Promega) as described previously [40]. For NCAG assays, samples were digested by NUDT5 in the NCAG buffer as follows. Samples were treated with NUDT5 at a final concentration of 400 nM and a final volume of 50 μL. These samples were incubated for 20 minutes at room temperature, then,10 μL of the samples were added to a 384 well microplate with the same volume of AMP-Glo Reagent 1 and incubated for one hour at room temperature. After incubation, 20 μL of AMP-Glo Detection Solution (prepared by mixing AMP-Glo Reagent 2 and Kinase-Glo^®^ in a 1:100 ratio) was then added to each well and incubated for one hour. Luminescence signals were then read using SYNERGY 4 plate reader (Biotek Instruments, Inc). For all NCAG assays unless otherwise stated, background luminescence signals were obtained from reactions evaluated by the NCAG assay before cleavage by ADP-ribosyl-acceptor hydrolases. The background signals were then subtracted from all samples and luminescence was converted to ADPR concentration based on a standard curve. This background subtraction allows for observed signal changes to be attributed solely to the digestion by ADP-ribosyl-acceptor hydrolases.

The reversal of MARylation was evaluated by treating 2 μM TARG1, ARH3, and PARG to Asp/Glu-MARylated or Ser-MARylated substrates for 1 hour at 37°C in a NCAG buffer. The activity of NUDT5 against isolated protein-free PAR chains was evaluated by incubating 38 μM of 7 mer PAR (prepared and calculated as described above) with 2 μM NUDT5 or 2 μM PARG for 1 hour at room temperature. The PAR sample was also tested with only the AMP-Glo assay reagents, and this control signal was used for the background subtraction of the NCAG assays to evaluate effects, if any, of the NUDT5 digestion step added in the NCAG. Then, the NCAG assays were used to measure the protein-free ADP-ribose released by these enzymes as described above, with a final volume of 37.5 μL used for the NUDT5 digestion.

To measure the kinetics of PARG, NCAG assays were performed by making 150 μL of master mix at desired concentrations of PARG and PARylated PARP1 at room temperature in the NCAG buffer. Control reactions before the addition of PARG were collected as the background signals and then reactions were started with the addition of PARG. At each time point of the time-course experiments, 20 μL of samples were collected and boiled for 1 minute to quench reactions. Then, the NCAG assay was used to measure the protein-free ADP-ribose released by these enzymes as described above, with final volumes of 50 μL.

The concentration of PARG-cleavable ADPR units in PARylated PARP1 was calculated through complete digestion with saturating conditions of PARG (2 μM) for 2 hours at room temperature in the NCAG buffer. ADPR concentration was then determined through the NCAG.

### Statistics

All AMP-Glo assays were done in a 384 well microplate in triplicate as described above. For all NCAG assays, standard deviations were calculated from triplicates and used for error bars in Figs 3–5. A Michaelis-Menton equation was used to calculate *K*_m_ and *K*_cat_ in Fig 5.

## Results and discussion

### NUDT5 selectively cleaves protein-free mono-ADP-ribose

To selectively monitor the release of protein-free ADP-ribose using the AMP-Glo assay, an enzyme that selectively and efficiently cleaves protein-free ADP-ribose, but not protein-bound or polymeric ADP-ribose, into AMP is required (Fig 1). The cleaved AMP will be subsequently quantified using the AMP-Glo assay that measures the AMP concentrations [34]. Furthermore, the enzyme should not cleave small molecules that are structurally similar to ADP-ribose, such as NAD^+^.

**Fig 2:**
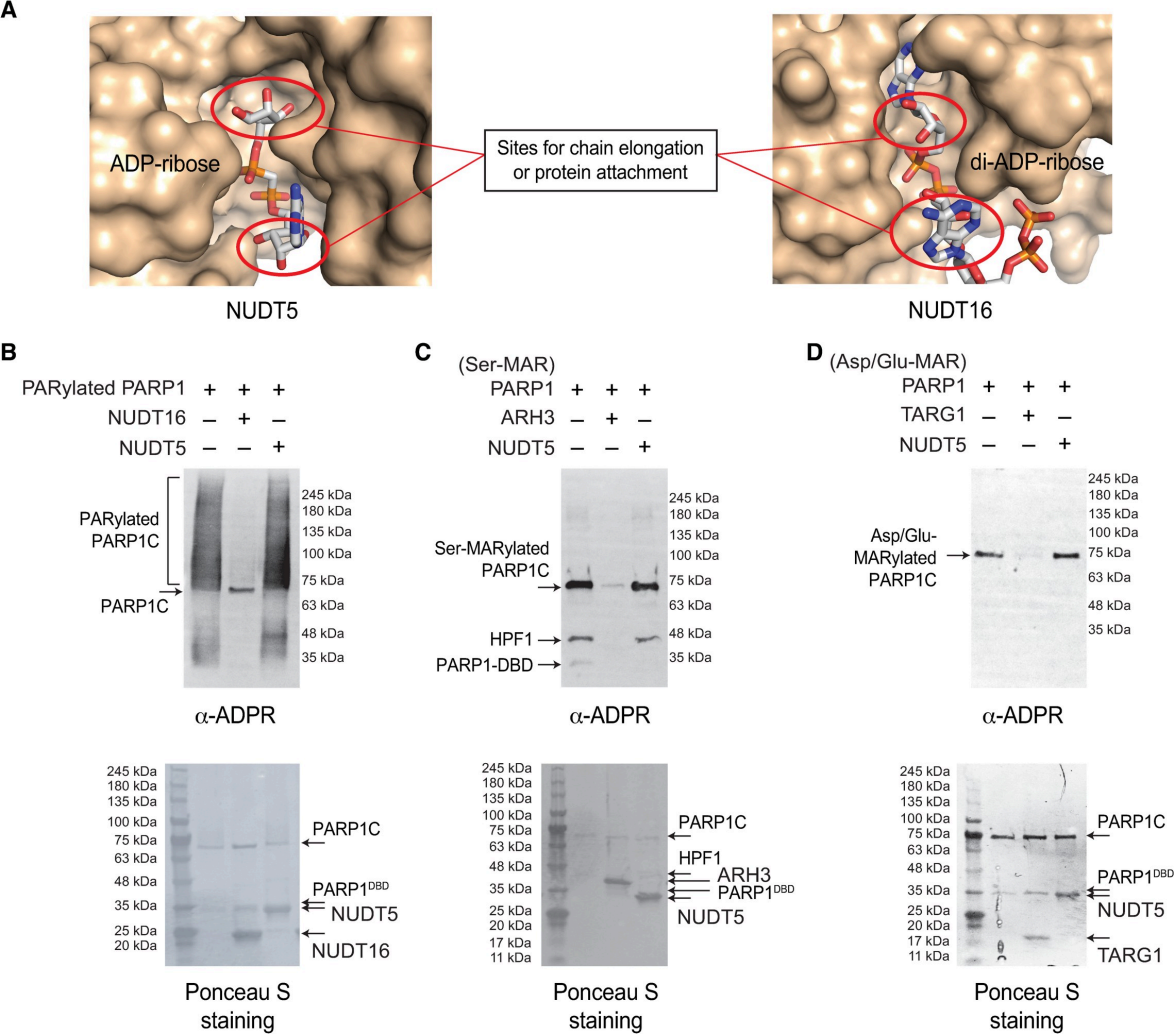
NUDT5 selectively cleaves protein-free ADP-ribose, but not protein-bound poly- and mono-ADP-ribosylations. A. NUDT5 is unable to bind to the protein-bound mono-ADP-ribosylation or poly(ADP-ribose). The sites for chain elongation or protein attachment are blocked in NUDT5 but are open in NUDT16. The substrate-binding pocket for NUDT5 and NUDT16 are superimposed and the orientations of ADP-ribose binding are shown. B. NUDT5 is unable to cleave the protein-bound PARylation. PARylated PARP1 was treated with recombinant human NUDT5 (5 μM) and NUDT16 (5 μM). While NUDT16 efficiently reverses the protein-bound PARylation, NUDT5 shows little-to-no PAR hydrolysis activity. C-D. NUDT5 is unable to cleave the protein-bound mono-ADP-ribosylations. Ser- and Asp/Glu-MARylated PARP1 substrates (see the “Materials and Methods”) were digested with 5 μM of each human ARH3 and NUDT5 (in panel C) and TARG1 and NUDT5 (in panel D), respectively. NUDT5 shows little-to-no MAR hydrolase activity, whereas ARH3 and TARG1 effectively reverse Ser- and Asp/Glu-MARylated PARP1 substrates, respectively.

Nudix family of phosphodiesterases hydrolyze ADP-ribose into AMP and ribose-5-phosphate [41–43]. For example, NUDT16 is able to digest both protein-bound ADP-ribosylations and protein-free ADP-ribosylations [44]. Among the Nudix family enzymes, NUDT5 drew our attention due to its unique substrate-binding mode (Fig 2A). These two enzymes share structural similarity in the core Nudix fold. However, when structures of NUDT5–ADPR complex (PDB ID: 2DSC) [45] and NUDT16–di-ADPR complex (PDB ID: 6B09) [44] were superimposed, surprisingly, the orientation of ADP-ribose bound to the enzyme is completely opposite.

In NUDT16, the sites for chain elongation (2′-OH of ribose′ or 1″-OH of terminal ribose″) or protein attachment (1″-OH of terminal ribose″) are widely open to solvent (Figs 2A and S1), allowing this enzyme to cleave PARylation in addition to ADP-ribose (Fig 2B) [44]. In contrast, ADP-ribose is bound in an opposite orientation in NUDT5 (Fig 2A). In this orientation, the access to both 2′-OH of the ribose′ and 1″-OH of the terminal ribose″ is effectively blocked (Fig 2A), and consequently the entrance of ADP-ribose chains or protein-bound MARylation to the NUDT5’s active site is prohibited. Consistent with this hypothesis, NUDT5 is unable to cleave PARylation in PARylated PARP1 substrates, while NUDT16 efficiently cleaves PARylation (Fig 2B).

Next, we tested the NUDT5’s substrate specificity to mono-ADP-ribosylated protein substrates (Fig 2C and 2D). To this end, we generated mono-ADP-ribosylated PARP1 substrates. Since PARG is unable to cleave the last ADP-ribose unit attached to target proteins [46], PARG was treated to PARylated PARP1 to generate MARylated protein substrates. HPF1 is required to switch the site specificity of PARP1 from Asp/Glu to Ser [47]. To specifically ADP-ribosylate PARP1 at Asp/Glu or Ser, we PARylated PARP1 in the absence and presence of HPF1, respectively. To monitor the level of ADP-ribosylation, western blotting was performed using AF1521-Fc [37], an anti-pan-ADPR antibody, that recognizes ADP-ribosylations on proteins.

As shown in Fig 2C and 2D, NUDT5 shows little-to-no hydrolysis of both Ser- and Asp/Glu-MARylated PARP1 substrates, whereas ARH3 [48] and TARG1 [49] specifically and efficiently reversed Ser- and Asp/Glu-MARylation, respectively. Collectively, these results demonstrate that NUDT5 is unable to cleave protein-bound poly- or mono-ADP-ribosylations.

### Establishment of NUDT5-Coupled AMP-Glo (NCAG) assay

**Fig 3:**
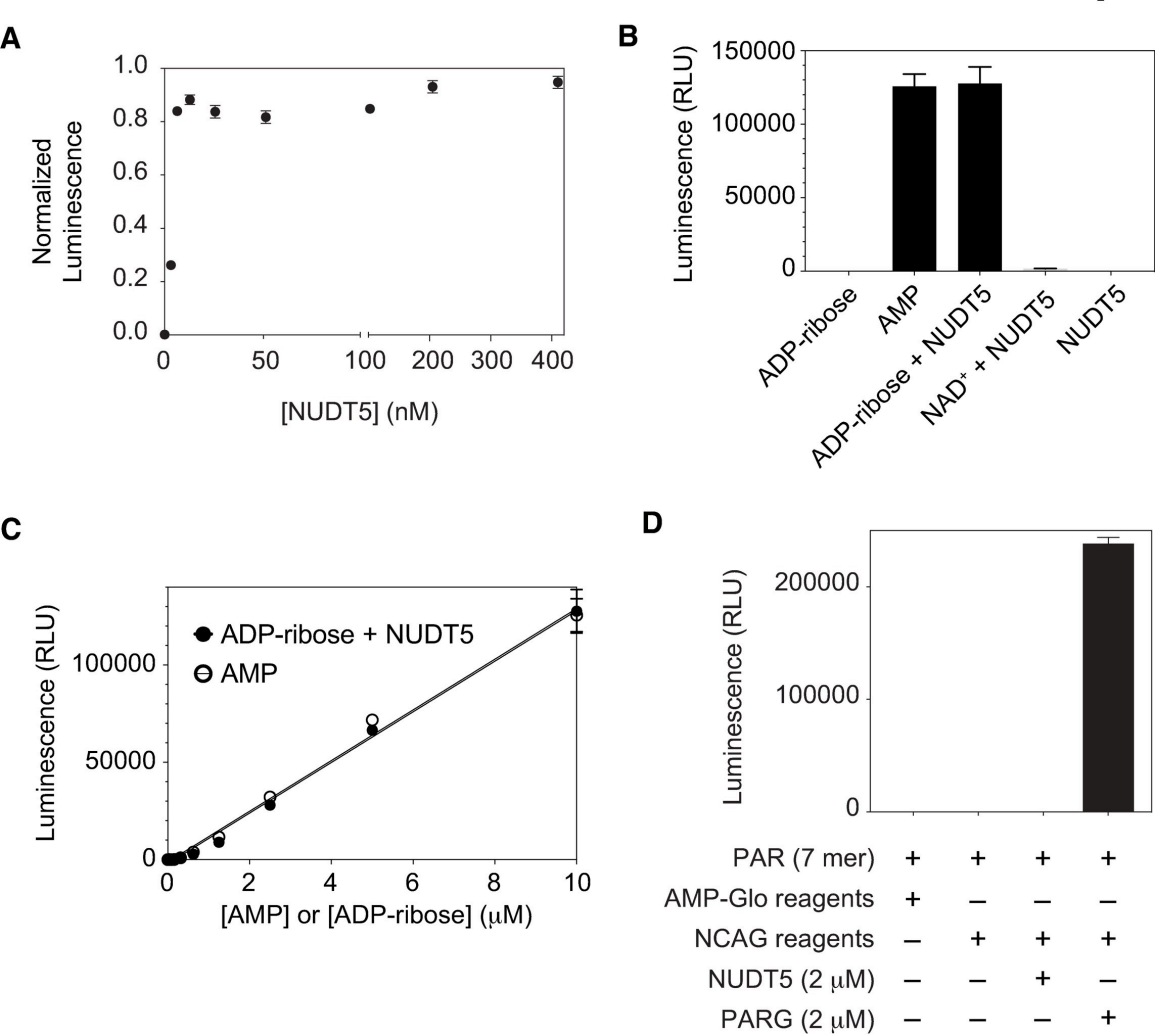
The NUDT5-coupled AMP-Glo (NCAG) assay effectively and selectively monitors the protein-free mono-ADP-ribose. A. NUDT5 titration against 10 μM ADPR. The luminescence signals from NUDT5-mediated digestion of ADPR are normalized with respect to that from 10 μM AMP. B. Comparison of NCAG luminescence signals from different types of substrates. Experiments were done using 10 μM of all substrates and 400 nM NUDT5. C. A standard curve of ADPR and AMP in the NCAG assay using 400 nM NUDT5. Standard deviations from triplicates were calculated for all NCAG assays and are shown as error bars. D. NUDT5 is unable to cleave protein-free poly(ADP-ribose) chains. Isolated PAR chains (7 mer, 38 μM) were assayed using the NCAG assay after NUDT5 (2 μM) and PARG (2 μM) digestions. A control reaction with the basic NCAG reagents (AMP-Glo reagents and 400 nM NUDT5) did not show signal increase in comparison to that with only AMP-Glo reagents. The AMP-Glo-only readout was used for background subtraction in panel D.

Next, we tested whether NUDT5 can efficiently cleave ADP-ribose into AMP. NUDT5 was titrated into 10 μM ADP-ribose and then the luminescence from the released AMP was measured using the AMP-Glo assay. The luminescence starts being saturated at NUDT5 concentration above ~ 10 nM (Fig 3A). However, to maximize the cleavage efficiency, 400 nM NUDT5 was selected for use in this assay, which generated ~ 97% of luminescence, with respect to that for an equimolar AMP (Fig 3A).

ADP-ribosyl transferases, such as PARPs, use β-NAD^+^ as a substrate. Since NAD^+^ has a similar structure to ADPR, this raises the possibility that unreacted residual β-NAD^+^ in ADP-ribosylated substrates may generate high background signal. This off-target effect against NAD^+^ would be an important factor in our NCAG assay, as a complete removal of NAD^+^ from ADP-ribosylation reactions can be time-consuming and very difficult. As seen in Fig 3B, NUDT5 showed nearly no NAD^+^ cleavage. In contrast, snake venom phosphodiesterase (svPDE) showed efficient cleavage (S2 Fig).

A standard curve was generated using our NUDT5-coupled AMP-Glo (NCAG) assay for ADP-ribose and compared to that for AMP (Fig 3C). NUDT5-depedent ADP-ribose cleavage was highly effective in all tested range (0 ~ 10 μM) (Fig 3C), and the results from the NCAG assay (for ADP-ribose) are nearly identical to those from AMP (Fig 3C). Together, these findings imply that NUDT5 can selectively digest the protein-free mono-ADP-ribose and our NCAG assay can effectively monitor the release of ADP-ribose by ADP-ribosyl-acceptor hydrolases.

Finally, we tested whether NUDT5 can digest the protein-free poly(ADP-ribose) chains. Control reactions containing the basic NCAG assay reagents (AMP-Glo reagents and 400 nM NUDT5) did not increase the background signal, in comparison to those containing only the AMP-Glo assay reagents (Fig 3D). Isolated protein-free PAR chains (7 mer) were not cleaved by NUDT5, whereas PARG efficiently cleaves PAR chains. Taken together, these results support that the NCAG assay can selectively monitor the protein-free mono-ADP-ribose in solution.

### Monitoring of the reversal of residue-specific mono-ADP-ribosylations by ADP-ribosyl-acceptor hydrolases

It has been shown that ARH3 and TARG1 can specifically reverse Ser- and Asp/Glu-MARylation, respectively [48,49]. Therefore, as a *proof-of-concept* of the NCAG assay, we tested whether the NCAG assay can be used to detect the deMARylation activity of site-specific ADP-ribosyl-acceptor hydrolases, ARH3 and TARG1. For all samples, the background signals from control reactions without ADP-ribosyl-acceptor hydrolases were subtracted and thus observed signal changes are attributed to the digestion by ADP-ribosyl-acceptor hydrolases.

**Fig 4:**
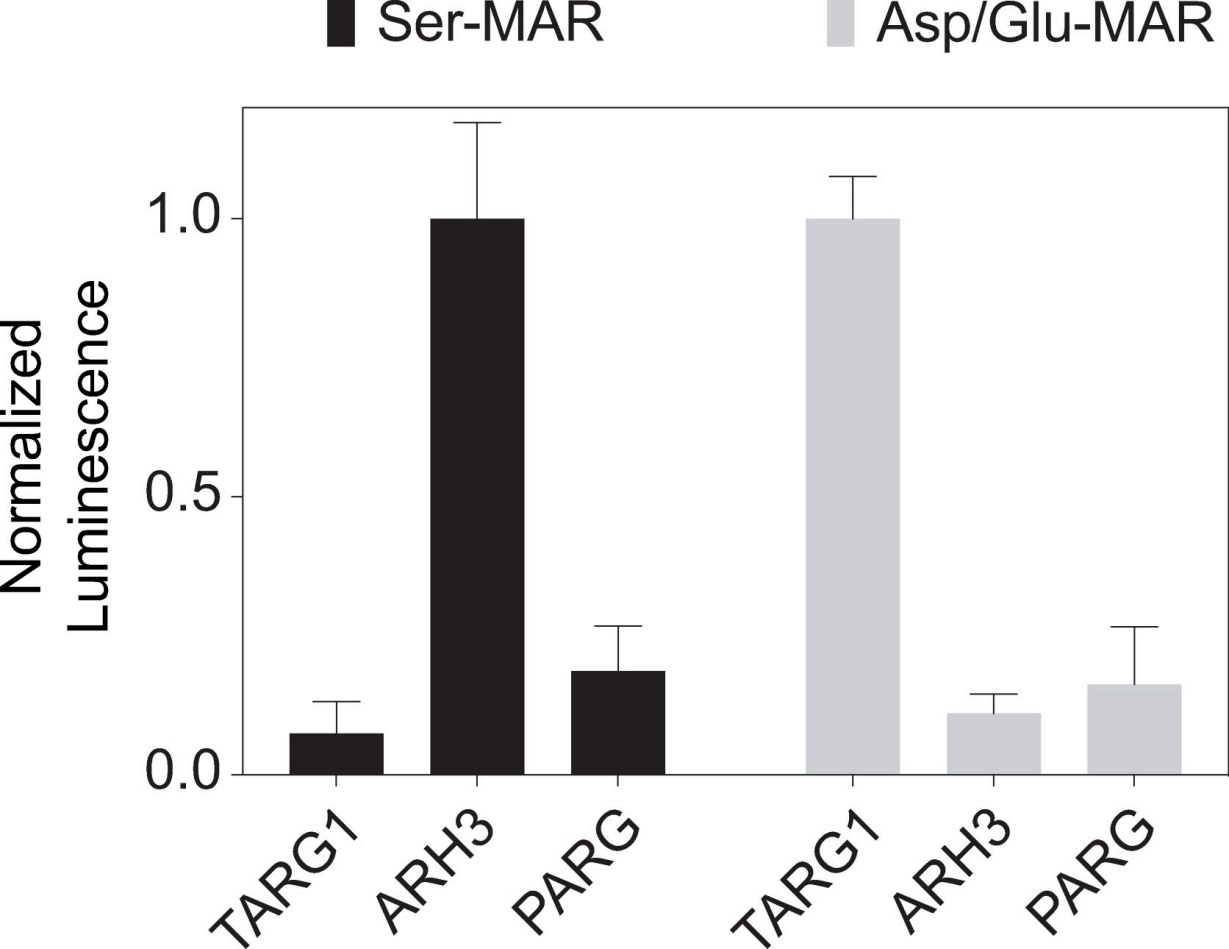
Monitoring of the reversal of residue-specific mono-ADP-ribosylations by ADP-ribosyl-acceptor hydrolases using the NCAG assay. The Ser- and Asp/Glu-MARylated PARP1 substrates (see the “Materials and Methods”) were digested with 2 μM TARG1, ARH3, and PARG, and released ADP-ribose was measured using the NCAG assay. While PARG shows limited hydrolysis of MARylated substrates, ARH3 and TARG1 specifically reverse the Ser- and Asp/Glu-MARylation, respectively. The luminescence signals from different enzymes were normalized with respect to that from ARH3 (in Ser-MAR) and TARG1 (in Asp/Glu-MAR), respectively. Standard deviations from triplicates were calculated for all NCAG assays and are shown as error bars.

As shown in Fig 4, the NCAG assay successfully detected the specific digestion of Ser-MARylation by ARH3, but not by TARG1 and PARG. Similarly, Asp/Glu-MARylation was efficiently digested by TARG1, but not by ARH3 and PARG. Together, these data support that the NCAG assay can be used to measure the activity of residue-specific ADP-ribosyl-acceptor hydrolases that release ADP-ribose as a product.

### A kinetic analysis of the exo-glycohydrolase activity of PARG

**Fig 5:**
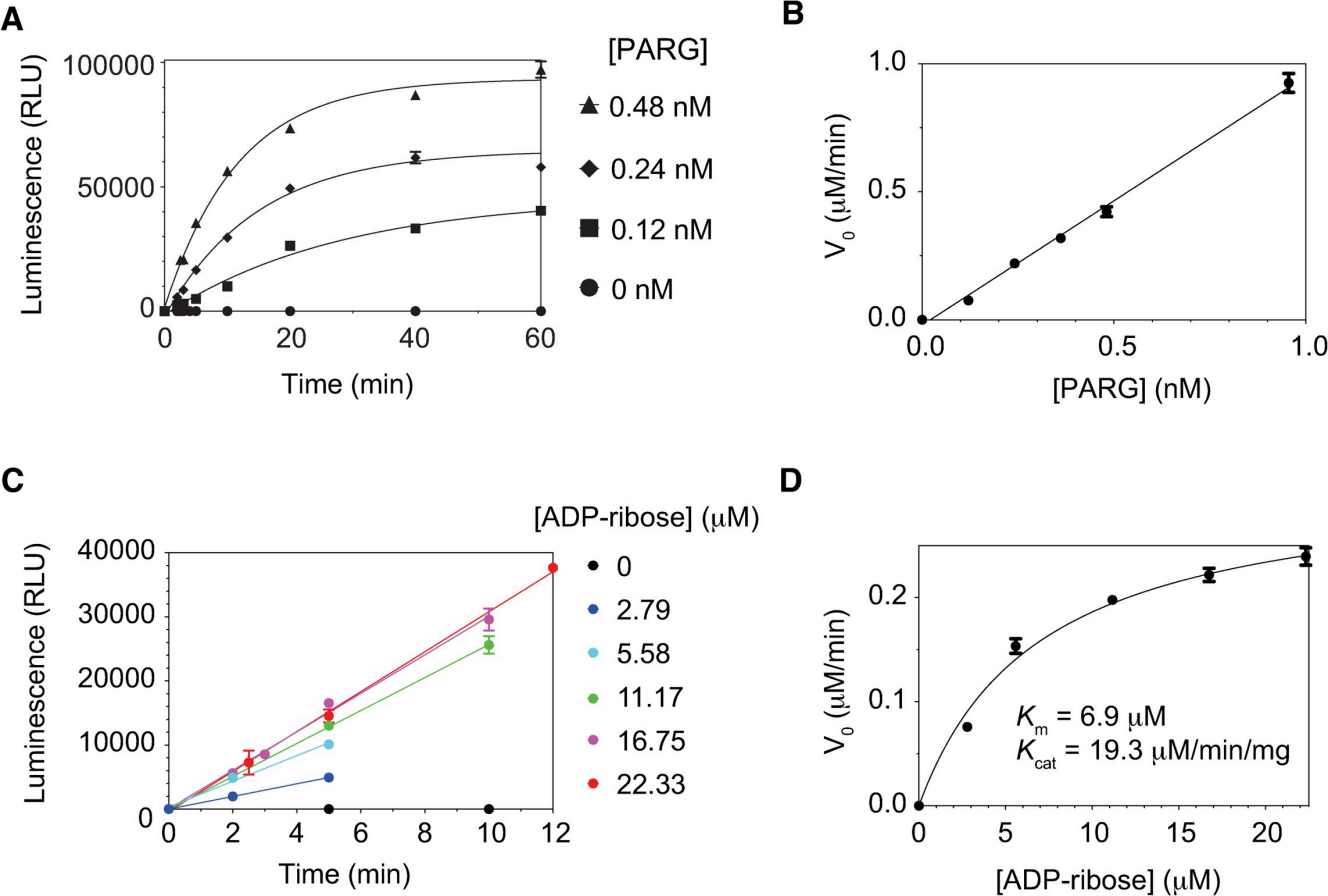
A kinetic analysis of the exo-glycohydrolase activity of PARG using the NCAG assay. A-B. A time-dependent and dose-dependent monitoring of the exo-glycohydrolase activity of PARG by the NCAG assay. 16.75 μM of ADP-ribose (cleavable ADP-ribose units in PARylated PARP1) was treated with increasing concentration of PARG. The initial velocities for each PARG concentration were calculated using the early and linear portions and plotted as a function of the PARG concentration in panel B. C-D. Michalis-Menton kinetics for the exo-glycohydrolase activity of PARG. The initial velocities were calculated at different concentrations of substrates (in panel C) and plotted as a function of substrate concentrations (in panel D). Standard deviations from triplicates were calculated for all NCAG assays and are shown as error bars.

It has been shown that PARG has both endo- and exo-glycohydrolase activity that cleaves the internal and terminal sites of PAR, respectively [18–20]. We tested whether we can selectively monitor the exo-glycohydrolase activity of PARG that releases ADP-ribose. PARylated PARP1 substrates were desalted to minimize the residual NAD^+^. Consistently, this substrate showed little-to-no background signal (Fig 5A), confirming that NUDT5 selectively digests the protein-free ADP-ribose, but not protein-bound ADP-ribosylation and unreacted NAD^+^.

The total concentration of cleavable ADPR units in PARylated PARP1 was calculated through a complete digestion of PARylated PARP1 with excess amount of PARG (2 μM) using the ADP-ribose standard curve (Fig 3C). At a near-saturating concentration of ADP-ribose (16.75 μM), PARG showed a dose-dependent and time-dependent increase in luminescence signal (Fig 5A). As expected, the initial velocities show a linear increase as a function of PARG concentration (Fig 5B).

Next, to gain the kinetic parameters of the exo-glycohydrolase activity of PARG, similar experiments were performed by varying the substrate concentrations at a fixed concentration of PARG (0.24 nM). The increase of substrate concentrations leads to the saturation of the initial velocity (Fig 5C). By fitting to a Michaelis-Menton equation, *K*_m_ and *K*_cat_ were calculated as 6.92 μM and 19.3 μM/min/mg, respectively (Fig 5D). Taken together, these findings support that the NCAG assay can efficiently and accurately measure the release of protein-free ADP-ribose by PARG and can be broadly used for various ADP-ribosyl-acceptor hydrolases.

### Concluding remarks

Although ADP-ribosylation has been proven as a key PTM that is responsible for a variety of cellular processes, the development of tools to quantitatively study the reversal of different types of ADP-ribosylations has been lagging. A protein-free ADP-ribose is the final reaction product in all ADP-ribosyl-acceptor hydrolases. Despite the growing interests and emerging biomedical importance of ADP-ribosylation removal, assays to study these erasers of ADP-ribosylations remain complex or cumbersome.

In this study, we have established a novel NCAG assay platform, in which NUDT5 selectively converts the protein-free monomeric form of ADP-ribose into AMP. Although the NCAG assay adopts an enzyme-coupled indirect measurement of ADP-ribose, this new assay provides a high sensitivity (down to low nanomolar concentrations of ADP-ribose) with a remarkable signal-to-noise ratio in the context of complex samples that include multiple proteins, residual NAD^+^, and different types of ADP-ribosylations, including protein-bound and protein-free PAR and MAR.

Notably, *K*_m_ and *K*_cat_ values obtained from the NCAG assay (Fig 5D) are comparable to published results (5.8 μM and 21 μM/min/mg, respectively) that were measured using the ^32^P-labeled PAR substrates [22]. This supports that the NCAG assay accurately reflects the kinetics of PAR hydrolysis by PARG. Although PARG has both exo- and endo-glycohydrolase activities [33,50], the balance between these two activities appears to be regulated by the ratio between PAR and PARG [50]. Our kinetic analysis using the NCAG assay suggests that the exo-glycohydrolase function of PARG is predominant in experimental conditions used in this and previous study [22].

The AMP/Glo assay has been used to detect AMP *in vitro* and in cell extracts [34,40]. Our data clearly demonstrated that the NCAG assay can selectively detect nanomolar concentration of protein-free ADP-ribose *in vitro* and suggest this NCAG assay can be used as an effective ADP-ribose sensor both *in vitro* and in cell extracts. Finally, this NCAG assay was developed in a multi-well format and therefore is suitable for high-throughput screening of small-molecule probes that specifically target the biomedically important ADP-ribosylation reversal enzymes, such as PARG [51], ARH3 [35,52], ARH1 [26,27], MacroD1/D2 [23,24], TARG1 [49], and SARS-CoV-2 Mac1 [53].

## Supporting information

S1 Fig Sites for the chain elongation or protein attachment in ADP-ribosylations. (PDF)

S2 Fig The snake venom phosphodiesterase (svPDE) efficiently cleaves NAD^+^. 10 μM NAD^+^ was treated with increasing concentrations of svPDE and the luminescence signals were measured using the AMP-Glo assay. The luminescence signals from the svPDE-mediated digestion of NAD^+^ were normalized to those from 10 μM AMP.
(PDF)

S1 Raw images Supporting images for Fig 2. Immunoblotting and membrane staining with respective loading controls showing the molecular size marker.
(PDF)
