# Supplementary material for: Selective monitoring of the protein-free ADP-ribose released by ADP-ribosylation reversal enzymes
Source: PLoS One. 2021 Jun 30;16(6):e0254022. doi: 10.1371/journal.pone.0254022 (PMC8244878; doi:10.1371/journal.pone.0254022)
Supplement: S1 Fig — (PDF) [file pone.0254022.s001.pdf]

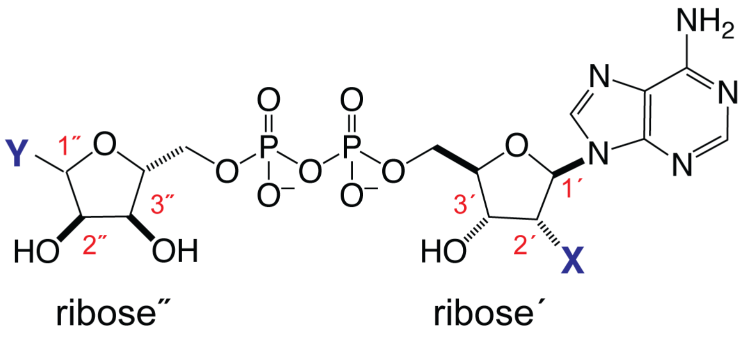

Y = OH
or ADP-ribose
or protein

X = OH
or ADP-ribose

S1 Fig. Sites for the chain elongation or protein attachment in ADP-ribosylations.
