# Supplementary material for: Selective monitoring of the protein-free ADP-ribose released by ADP-ribosylation reversal enzymes
Source: PLoS One. 2021 Jun 30;16(6):e0254022. doi: 10.1371/journal.pone.0254022 (PMC8244878; doi:10.1371/journal.pone.0254022)
Supplement: S2 Fig — 10 μM NAD+ was treated with increasing concentrations of svPDE and the luminescence signals were measured using the AMP-Glo assay. The luminescence signals from the svPDE-mediated digestion of NAD+ were normalized to those from 10 μM AMP. (PDF) [file pone.0254022.s002.pdf]

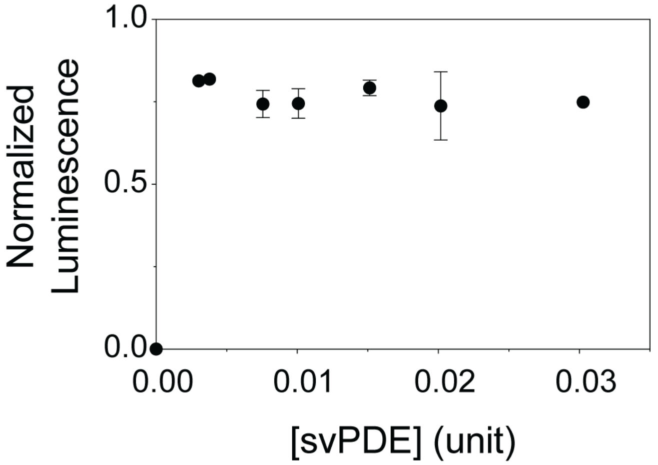

S2 Fig. The snake venom phosphodiesterase (svPDE) efficiently cleaves NAD⁺. 10 μM NAD⁺ was treated with increasing concentrations of svPDE and the luminescence signals were measured using the AMP-Glo assay. The luminescence signals from the svPDE-mediated digestion of NAD⁺ were normalized to those from 10 μM AMP.
