# Supplementary material for: Selective monitoring of the protein-free ADP-ribose released by ADP-ribosylation reversal enzymes
Source: PLoS One. 2021 Jun 30;16(6):e0254022. doi: 10.1371/journal.pone.0254022 (PMC8244878; doi:10.1371/journal.pone.0254022)
Supplement: S1 Raw images — Immunoblotting and membrane staining with respective loading controls showing the molecular size marker. (PDF) [file pone.0254022.s003.pdf]

Supporting Images for Figure 2B

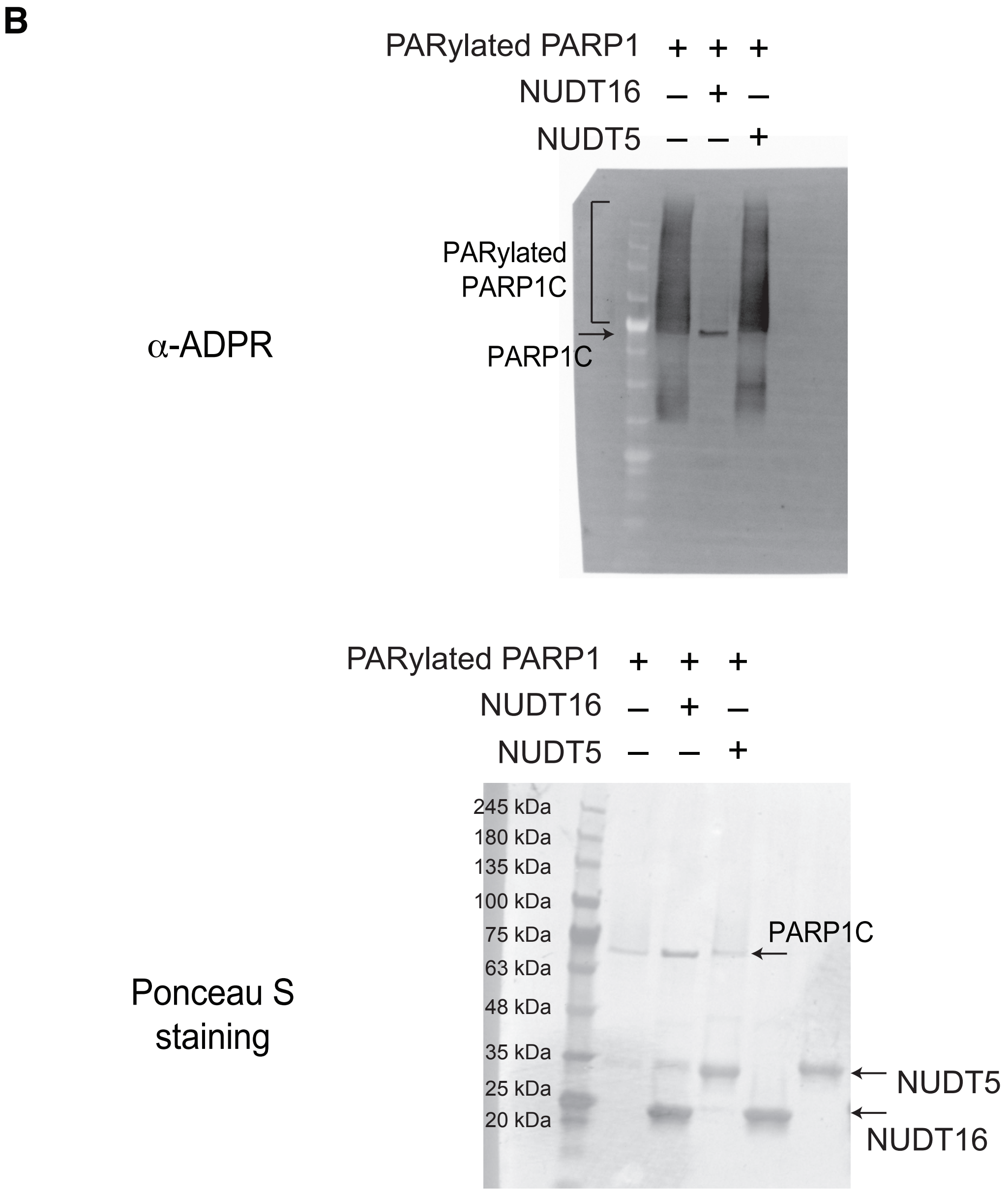

Images showing poly(ADP-ribose) hydrolysis by NUDT16 and NUDT5 monitored by western blotting and ponceau S membrane staining.

Supporting Images for Figure 2C

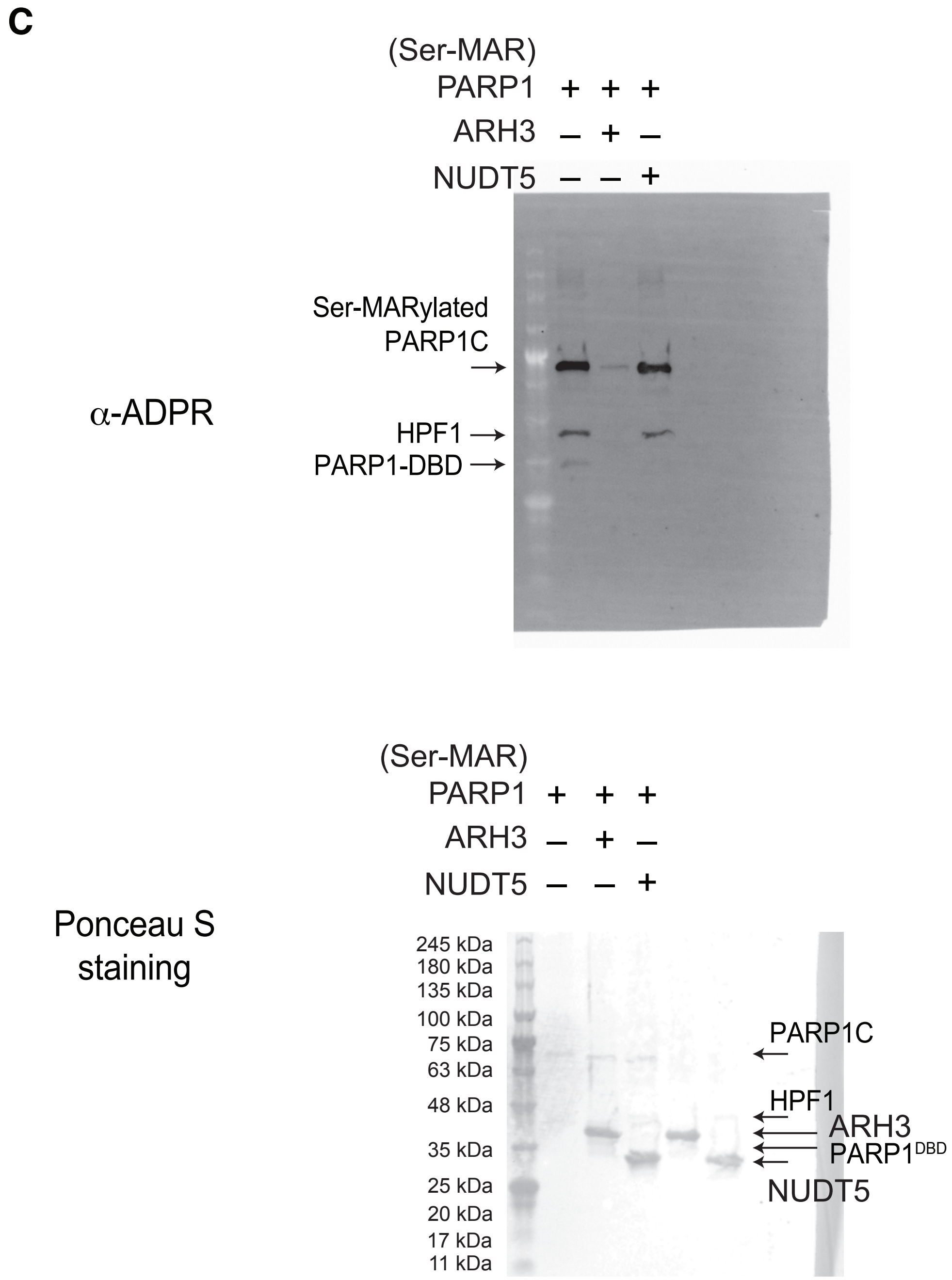

Images showing serine mono-ADP-ribosylation hydrolysis by ARH3 and NUDT5 monitored by western blotting and ponceau S membrane staining.

Supporting Images for Figure 2D

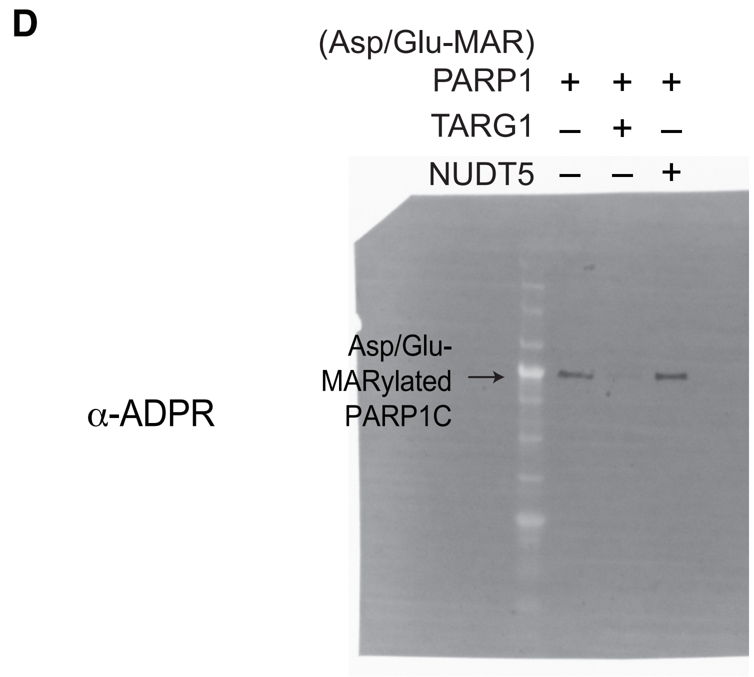

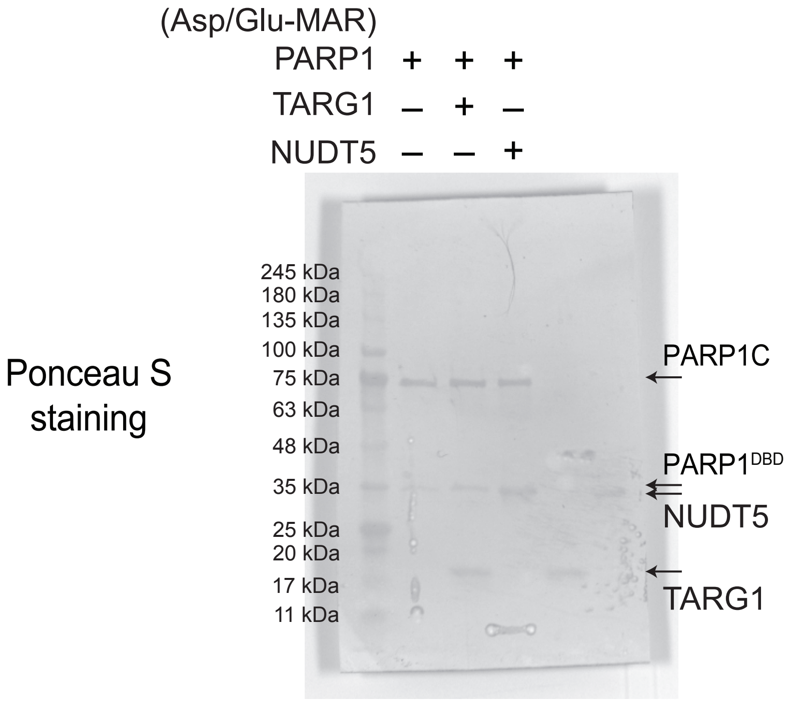

Images showing Asp/Glu mono-ADP-ribosylation hydrolysis by TARG1 and NUDT5 monitored by western blotting and ponceau S membrane staining.
